# Maternal Extra Virgin Olive Oil Supplementation Enhances Offspring Immune Function: A Preclinical Study

**DOI:** 10.3390/ijms26167946

**Published:** 2025-08-18

**Authors:** Sonia Zhan-Dai, Blanca Grases-Pintó, Adriana García-Vara, Ruth Ferrer, Raquel Martín-Venegas, Rosa M. Lamuela-Raventós, Margarida Castell, Francisco J. Pérez-Cano, Anna Vallverdú-Queralt, Maria J. Rodríguez-Lagunas

**Affiliations:** 1 Department of Biochemistry and Physiology, Faculty of Pharmacy and Food Science, University of Barcelona, 08028 Barcelona, Spain; soniazhan@ub.edu (S.Z.-D.); blancagrases@ub.edu (B.G.-P.); adriana.garcia@ub.edu (A.G.-V.); rutferrer@ub.edu (R.F.); raquelmartin@ub.edu (R.M.-V.); margaridacastell@ub.edu (M.C.); 2 Institute of Nutrition and Food Safety (INSA-UB), University of Barcelona, 08921 Santa Coloma de Gramenet, Spain; lamuela@ub.edu (R.M.L.-R.); avallverdu@ub.edu (A.V.-Q.); 3 Department of Nutrition, Food Science and Gastronomy, Faculty of Pharmacy and Food Science, University of Barcelona, 08028 Barcelona, Spain; 4 CIBER Physiopathology of Obesity and Nutrition (CIBEROBN), Institute of Health Carlos III, 28029 Madrid, Spain

**Keywords:** extra virgin olive oil, maternal supplementation, offspring, immune system

## Abstract

Maternal diet influences offspring development, immune function, and intestinal health. This study investigates the effects of maternal supplementation with a key component of the Mediterranean Diet, extra virgin olive oil (EVOO), on the immune health of offspring at the end of lactation. Lewis rat dams received either refined olive oil (ROO), EVOO, or water (REF) during gestation and lactation. Plasma immunoglobulin G2c (IgG2c) concentration was elevated in pups born to EVOO-supplemented mothers, indicating enhanced immune development. Histological analysis of the small intestine revealed more goblet cells in the EVOO group, indicating a potential positive effect on the intestinal barrier function. In vitro assays showed that EVOO metabolites did not display cytotoxicity and had improved barrier integrity under a stress stimulus. These findings suggest that maternal EVOO supplementation may have beneficial effects on immune and intestinal development and health in offspring.

## 1. Introduction

The immune system of the newborn is immature at birth, leading to significant deficiencies in acquired immunity, as evidenced by a lower concentration of plasma immunoglobulins (Ig) [1,2]. This acquired immune response is reduced due to a lack of contact with pathogens until birth, so the infant during the first days is mainly protected by its immature innate immunity and the vertically passive protection provided by its mothers through breast milk [3,4].

In line with this, it is also well known that the breastfeeding period is essential to continue the build-up of the immune system (IS) of the newborn after birth, as mother’s milk provides passive immunity to the infant, facilitates the establishment of the microbiota, and transfers active metabolites [5], stimulating the maturation of the infant IS.

The composition of breast milk is influenced by the exposome of the mother, which includes lifestyle, genetic, and environmental factors, such as the maternal diet [6,7,8]. These factors can impact the abundance of active milk components [9,10]. Thus, interventions aimed at improving nutrition during pregnancy and lactation can serve as a strategy to improve the properties of breast milk. In fact, previous studies involving maternal interventions with certain dietary components, both in preclinical and clinical settings, have demonstrated changes in the lipid and immune profiles of breast milk [9,10,11].

Extra virgin olive oil (EVOO) is considered one of the healthiest products from the Mediterranean region and serves as the primary fat source in this diet [12,13]. The beneficial properties of EVOO have been associated with components such as monounsaturated fatty acids, but especially with phenolic compounds [14,15], which make up to 1–2% and are considered minor components [13,16]. Among these, there is a diverse array of phenolic antioxidants, including phenyl alcohols, phenolic acids, secoiridoids, flavonoids, and lignans, highlighting the presence of tyrosol, hydroxytyrosol, oleuropein, and oleocanthal [17,18,19]. Several studies have shown that the phenolic compounds of EVOO have anti-inflammatory, anti-microbial, and antioxidant activities [15,20,21,22]. All these characteristics are associated with the prevention of neurodegenerative diseases, cancer, and rheumatic pathologies [23], apart from EVOO’s key role in cardiovascular diseases [24,25,26,27]. In addition, in previous studies, it has been observed that EVOO administration to dams during gestation and lactation increases IgA in breast milk [16], suggesting an additional immunomodulatory benefit in early life.

The vertical transmission of EVOO metabolites from the mother to the offspring in supplemented rats has recently been described [28]. In that study, homovanillic acid (HV), a derivative of hydroxytyrosol, was found in even higher concentrations in pups than in their respective mothers. One of the primary microbial metabolic transformations of hydroxytyrosol results in hydroxyphenylacetic acid (HFA), which was also detected at higher concentrations in both mothers and pups but was found in only minimal amounts in breast milk. In contrast, hippuric acid (HPP) was the microbial metabolite found with the highest concentration in breast milk [28].

In order to better understand the role of EVOO in the health of offspring, this preclinical study aimed to assess the impact of maternal EVOO supplementation during gestation and lactation on the immune system development of the offspring. Additionally, in vitro experiments were conducted to further understand the role of EVOO in the intestinal barrier.

## 2. Results

### 2.1. Body Weight and Morphometric Variables

Pups from three groups were born with a similar body weight (BW) (about 6.5–7 g), which increased gradually during the suckling period. After day 5 of life, pups whose mothers received ROO and EVOO supplementation during gestation and lactation slowed down their BW increase with respect to REF animals (Figure S1).

The lower BW in the ROO and EVOO groups resulted in a lower BMI at the end of suckling, but without affecting their Lee index. In addition, the naso-anal length of the EVOO group was lower than that of the REF group (Table 1).

With regard to the impact of maternal supplementation on offspring organ weights (Table 1), stomach weight increased in both oil-administered groups, and liver weight was slightly higher in the ROO group than in the EVOO group. The relative weights of the heart, kidney, spleen, and thymus were not modified due to maternal EVOO supplementation. Finally, an increase in small intestine length was observed in both the ROO and EVOO groups compared to REF animals. Additionally, small intestine weight was higher in the ROO group than in the other two groups.

**Figure 1:**
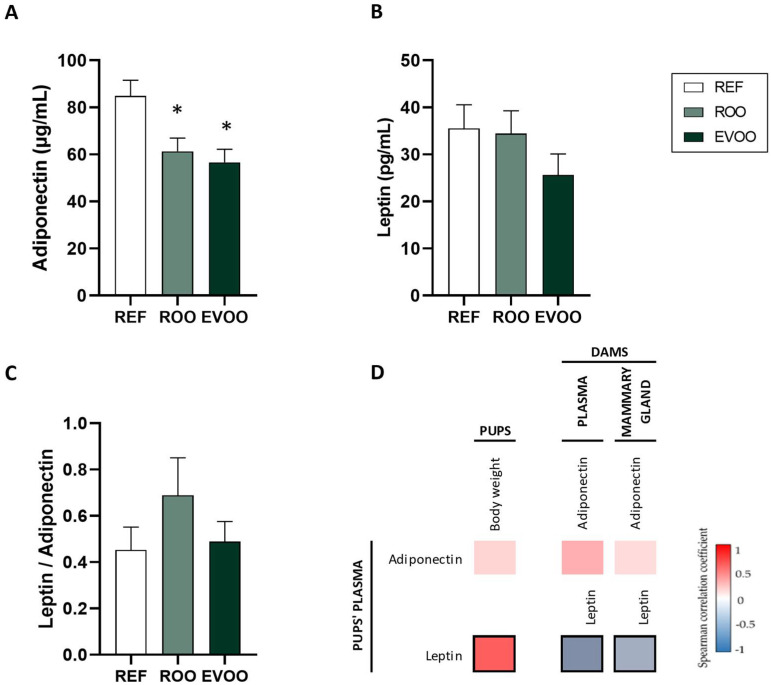
Plasma adiponectin and leptin levels at the end of the sucking period. Adiponectin (**A**) and leptin (**B**) levels and their ratio (**C**) in REF, ROO, and EVOO groups. Spearman correlations between pups’ plasma and their weight and between pups’ plasma and both dams’ plasma and mammary gland (**D**). Results are expressed as mean ± standard error of the mean (SEM) (n = 12–15 pups/group). Statistical differences: * *p* < 0.05 vs. REF group. Spearman’s correlation coefficient is represented in the heat map following the color in the legend. Bold frames represent correlations with statistical significance (*p* < 0.05).

To better understand the changes in BW, plasma leptin and adiponectin levels were studied. Both ROO and EVOO pups showed lower levels of adiponectin (Figure 1A) without changes in the leptin or leptin/adiponectin ratio (Figure 1B,C). Adiponectin levels in the pups did not correlate with their BW, nor with the adiponectin levels in the dams’ plasma or mammary glands (Figure 1D) [14]. However, the pups’ plasma leptin levels positively correlated with their BW and negatively correlated with the leptin concentrations in both the dams’ plasma and mammary glands.

Furthermore, concentrations of galectin-3 and 9, which play diverse roles in immune regulation, inflammation, and development, were also analyzed. The results do not show statistical differences due to the supplementation; however, Gal-9 concentrations with anti-inflammatory qualities tended to increase in the EVOO group with respect to REF animals (Figure S2).

No effect on the leukocyte count was observed in the pups due to the dams’ supplementation. However, some differences were found in erythrocyte count and hematocrit (HCT). ROO pups had an increase in erythrocyte counts and HCT with respect to the REF and EVOO groups (Table 2).

### 2.2. Immunoglobulin Profile in Plasma

**Figure 2:**
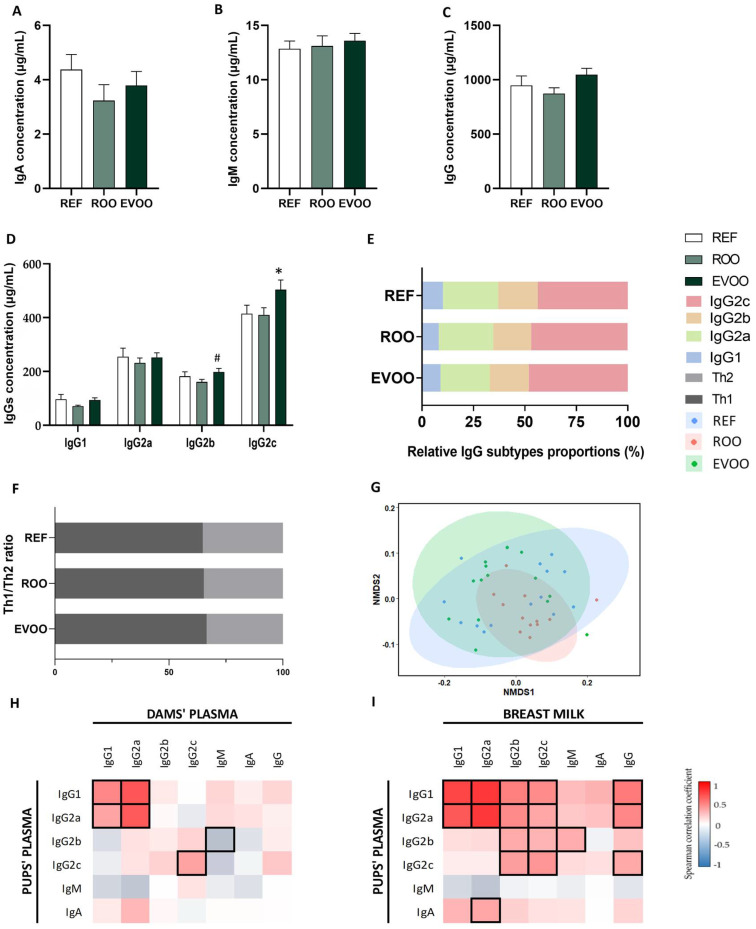
Effect of maternal supplementation on the Ig profile in pups’ plasma. Plasma IgA (**A**), IgM (**B**), and IgG (**C**) from REF, ROO, and EVOO groups. IgG subtypes (**D**) and their relative proportion (**E**). Analysis of the Th1/Th2 ratio at the end of suckling (**F**). Analysis of non-parametric multidimensional scaling (NMDS) for the Ig profiles based on the Bray–Curtis distance (**G**). Spearman correlations between dams’ plasma and pups’ plasma (**H**) and between breast milk and pups’ plasma (**I**). Results (**A**–**D**) are expressed as mean ± standard error of the mean (SEM) (n = 12–15). Statistical differences: * *p* < 0.05 vs. REF # *p* < 0.05 vs. ROO. The Spearman correlation coefficient is represented in the heat map following the color in the legend. Bold frames represent correlations with statistical significance (*p* < 0.05).

The plasma concentrations of IgA, IgM, and IgG isotypes (IgG1, IgG2a, IgG2b, and IgG2c) were quantified (Figure 2). Although no differences were observed between groups in IgA, IgM, or total IgG concentrations (Figure 2A–C), pups from the EVOO group showed a significant increase in IgG2c concentrations (Figure 2D) without affecting the overall IgG proportion (Figure 2E) or the Th1/Th2 ratio (Figure 2F). Moreover, the non-parametric multidimensional scaling (NMDS) Ig distribution did not display different clustering for Ig (Figure 2G). To further study the influence of the EVOO supplementation of the mothers on the pups, Spearman’s correlations between the Ig levels of the mothers and the pups were performed. IgG2c concentrations in the plasma of the pups were directly correlated with those of the mothers, both in plasma (Figure 2H) and in breastmilk (Figure 2I). Finally, Ig concentrations in MLN homogenates were also studied, but no differences due to maternal supplementation were found (Figure S3).

### 2.3. Intestinal Effects of EVOO

**Figure 3:**
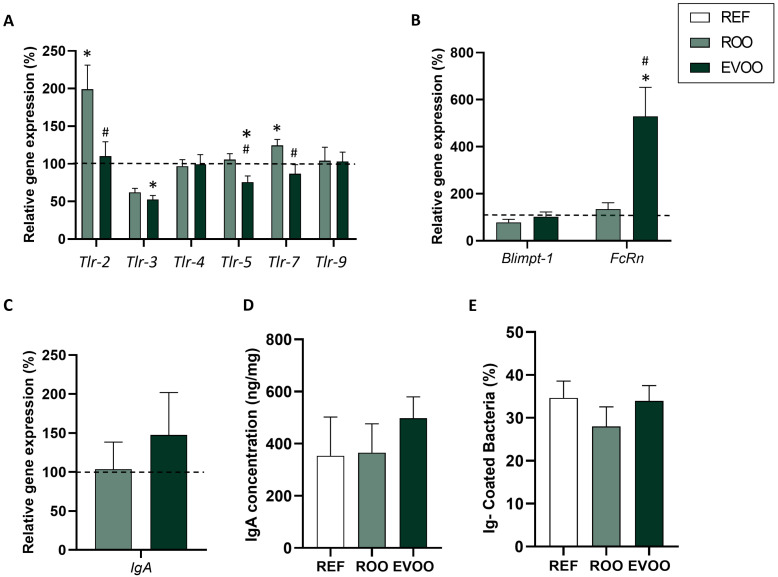
Gene expression of Toll-like receptors (TLRs) (**A**), maturation biomarkers (**B**), and IgA (**C**) at the end of the suckling period in the small intestine. Results are expressed with respect to the REF group, which corresponded to 100% of transcription (dashed line) (n = 9 pups/group). IgA concentration (**D**) in small intestine and Ig-coated bacteria (**E**) in cecal content. Results are expressed as mean ± standard error of the mean (SEM) (n = 18–36). Statistical differences: * *p* < 0.05 vs. REF group; # *p* < 0.05 vs. ROO group.

The gene expression of receptors involved in microbiota–immune system interactions, such as Toll-like receptors (TLR), was studied in the small intestines of pups at the end of the suckling period. A decrease in the expression of intestinal genes *Tlr-3* and *Tlr-5* in the EVOO group was found (Figure 3). The gene expression of some biomarkers of intestinal maturation—such as *Blimp-1*, a transcriptional factor involved in immune regulation and differentiation of plasma cells [29], and *FcRn*, a neonatal Fc receptor that is important for IgG transport across the intestinal epithelium [30]—was also analyzed. A marked increase in *FcRn* expression was detected in pups from the EVOO group (Figure 3B).

With regard to intestinal IgA gene expression (Figure 3C) and luminal IgA protein levels (Figure 3D), no differences were observed among groups. The proportion of bacteria bound to Ig in cecal samples was also analyzed at the end of the suckling period, and no differences were observed either (Figure 3E).

**Figure 4:**
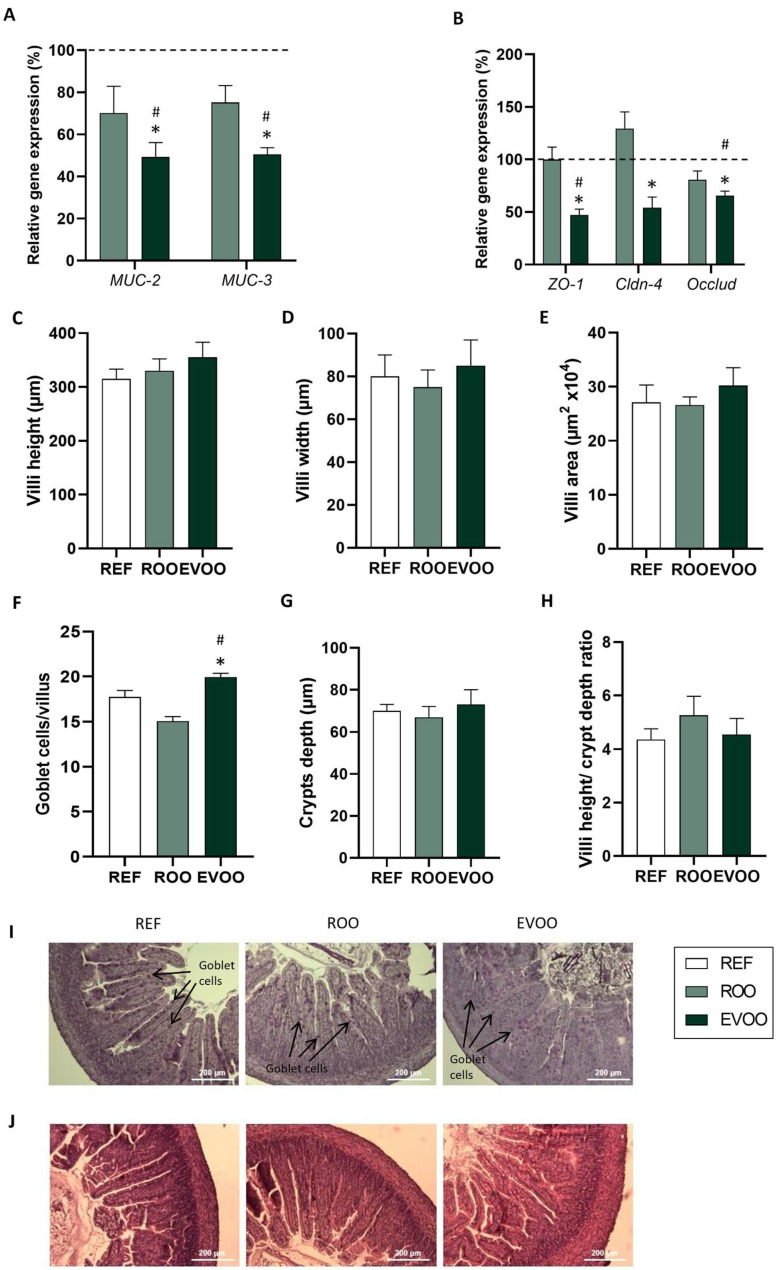
Effect of ROO and EVOO on the small intestine of the three groups of pups at the end of the suckling period. Gene expression of intestinal mucins (**A**) and tight junctions (**B**), calculated with respect to REF, which corresponded to 100% transcription (dashed line) (n = 9 pups/group). Height (**C**), width (**D**), area of the villi (**E**), number of Goblet cells/villi (**F**), crypt depth (**G**), and ratio of villi height–crypt depth (**H**) of the three groups. Results in (**C**–**H**) are expressed as mean ± standard error of the mean (SEM) (n = 18–36 pups/group). Statistical differences: * *p* < 0.05 vs. REF group; # *p* < 0.05 vs. ROO group. Representative images of histological sections of the medial intestine with periodic acid–Schiff (PAS) (**I**) and hematoxylin–eosin staining (**J**). Goblet cells with densely stained granules can be observed along the villi (**I**). Scale bar = 200 μm for 10×.

The expression of genes related to epithelial barrier function—such as mucins *Muc-2*, *Muc-3* (Figure 4A), or tight junction proteins, such as *Zo-1*, *Cldn-4*, and *Occlud* (Figure 4B)—was also evaluated. EVOO pups showed a lower intestinal gene expression in all of these molecules. The morphology of the small intestine was also evaluated, and no changes were observed in villus height, weight, or area or crypt depth (Figure 4C,D,E,G). Nevertheless, animals from the EVOO group exhibited a higher number of goblet cells in the small intestine than in the other two groups (Figure 4F).

### 2.4. In Vitro Effect of EVOO Metabolites on Caco-2 Cells

**Figure 5:**
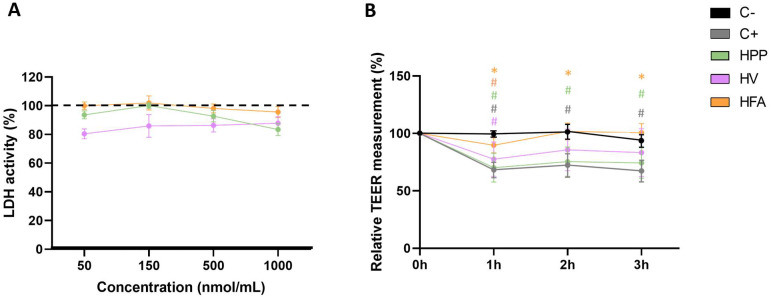
LDH activity as an indicator of cell membrane integrity (**A**). Measurement of TEER values in Caco-2 cells at different time points (1 h, 2 h, 3 h) during incubation with hippuric acid (HPP), homovanillic acid (HV), and hydroxyphenyl acetic acid (HFA) (**B**). Results are expressed as a percentage, with the value at time 0 set to 100%. Statistical differences: * *p* < 0.05 vs. C+. # *p* < 0.05 vs. C-. C-, control conditions (medium); C+, positive control (H_2_O_2_, 3 mM).

To better understand the effect of EVOO metabolites on the intestinal barrier, various assays using EVOO metabolites on Caco-2 cells were conducted. To assess the potential cytotoxicity of the three selected metabolites, HPP, HV, and HFA, the relative lactate dehydrogenase (LDH) activity was evaluated. None of the metabolites were toxic at the concentrations tested (Figure 5A).

Permeability assays revealed that HFA not only counteracted the disruptive effects of H_2_O_2_ but also showed an improvement in the barrier integrity compared to the positive control, confirmed by TEER (Figure 5B).

## 3. Discussion

EVOO is a key component of the Mediterranean Diet and stands out for its antioxidant, anti-inflammatory, and antimicrobial properties [13,15]. In previous studies, we also demonstrated that supplementation during gestation and lactation has a positive impact on maternal health [16] and results in the transfer of EVOO metabolites to offspring [28]. However, the effect of those metabolites on offspring health has not been described yet. In this study, we further explored the impact of the administration of EVOO to mothers during gestation and lactation on the offspring, focusing on the immune systems and intestinal health of lactating pups at the end of the lactation period.

The supplementation during gestation did not impact the birth weight of the pups. However, EVOO pups displayed lower body weight, together with a decrease in BMI, but without affecting the Lee index. These findings align with a study on mice where maternal EVOO consumption reduced offspring weight at birth and during adolescence, linked to metabolic benefits such as improved glucose homeostasis and lipid profiles [31]. These effects are likely influenced by the phenolic compounds in EVOO, such as hydroxytyrosol and oleuropein. Fasting glucose regulation seems to be crucial, as these compounds can improve insulin action and secretion, which is key for better glucose balance and preventing metabolic issues like obesity and type 2 diabetes [31,32,33].

Additionally, plasma adiponectin and leptin levels were measured to further investigate the metabolic effects of maternal EVOO supplementation. Adiponectin, which is known for its insulin-sensitizing and anti-inflammatory properties [34,35], was significantly reduced in the offspring, whereas leptin levels and the leptin–adiponectin ratio remained unchanged. While reduced adiponectin concentrations are generally linked to increased adiposity and metabolic disturbances, in our study, they were associated with lower body weight in the EVOO-exposed pups. This finding suggests that other regulatory mechanisms—potentially involving early-life programming of adipose tissue or altered milk composition—may more directly influence growth and energy homeostasis in this context. Other studies in sows and rats have shown that the maternal dietary fat source, particularly oleic acid from olive oil, modifies the milk fatty acid profile and correlates negatively with body weight gain in offspring by reducing food intake [36,37,38]. The lower body weight in the EVOO pups was not accompanied by changes in organ weight, indicating that the reduction does not reflect compromised health.

To explore the impact of EVOO on the immune system, plasma Ig concentration was analyzed. Total IgG, IgM, and IgA levels did not show significant changes due to maternal nutritional interventions. However, the mothers’ supplementation with EVOO increased the IgG2c concentration in plasma. Several studies report that the analog of IgG2c in mice has been associated with regulatory responses in the intestine and long-term immunity [39,40,41]. Therefore, the increase in IgG2c induced by maternal EVOO supplementation might contribute to enhanced systemic and intestinal immune regulation in offspring, potentially supporting long-term immune defense. The ROO group did not exhibit the same increase, suggesting that the phenolic compounds in EVOO may play a key role in this immune modulation.

Although no significant differences in plasma IgG2c concentration were observed in supplemented dams [16], we did find a direct correlation between the IgG2c levels in mothers and offspring. Additionally, a positive correlation was also observed between the IgG2c levels in maternal milk and the plasma IgG2c concentrations of the pups. This suggests that maternal IgG2c may influence the offspring’s immune profile. The transfer of IgG2c through breastfeeding could enhance their immune protection during early life, protecting them from pathogens [42]. This emphasizes the crucial role of maternal antibodies in the offspring’s immune system [43]. In fact, the evaluation of *FcRn* gene expression, which is responsible for the specific transport of IgG in the fetal and neonatal intestine and typically decreases at weaning [44], showed that *FcRn* expression is strongly increased by maternal EVOO supplementation. This overexpression is consistent with the increased levels of plasma IgG2c observed in pups. This finding aligns with other studies, which have demonstrated that *FcRn* overexpression can enhance the immune response in transgenic mice by extending the half-life of IgG [45,46]. Conversely, the ROO pups did not show this increase, reinforcing the idea that the specific phenolic compounds are driving this effect.

An increase in the length of the small intestine was also observed in the ROO and EVOO groups, whereas its weight was only increased in the ROO group and remained similar in the other two groups. Interestingly, an increase in intestinal length and weight in the mothers supplemented with EVOO was also observed [16], suggesting that the trophic effect of both ROO and EVOO may extend to both the mothers and their offspring. The role of EVOO metabolites on this effect should be further studied.

With regard to the intestinal histomorphometric analysis, the EVOO intervention led to an increase in the number of goblet cells compared to the other two groups. Goblet cells play a key role in maintaining the intestinal barrier, secreting mucins that form the mucus layer that protects the intestinal epithelium [47,48,49,50]. In contrast, the ROO group exhibited a lower number of goblet cells than the REF group, suggesting intestinal immaturity, resulting in diminished mucus production at the intestinal barrier. Although previous research has shown that compounds found in certain foods, like proanthocyanidins, can restore impaired intestinal barrier function by increasing the size and number of goblet cells, thereby enhancing MUC-2 mucin secretion [51,52], the specific effects of EVOO on goblet cells remain largely unexplored.

In our case, EVOO pups showed lower gene expression in both *Muc-2* and *Muc-3,* genes related to the mucus layer of the epithelial barrier. However, the higher number of goblet cells observed in these animals may compensate for this lower expression and might still maintain a protective response. Overall, new studies addressing the specific role of EVOO in mucin production are warranted. *ZO-1*, *Cldn-4*, and *Occlud* also showed lower gene expression. Similarly, a reduced expression of TLR genes was also observed, potentially contributing to immune homeostasis, as the lower expression of these genes limits excessive immune activation in the constant presence of microbes [53,54,55].

Since we observed the reduced gene expression of these tight junction proteins, we carried out in vitro experiments using selected metabolites from EVOO so that we could provide a better understanding of how EVOO affects the functioning of the intestinal barrier. The experiments did not show any negative effects, suggesting that the observed changes in gene expression might not translate into changes in protein expression.

Our in vitro experiments demonstrate that EVOO metabolites do not appear to have a negative effect on the intestinal barrier, as none of the metabolites assayed here were found to be cytotoxic. Specifically, HFA counteracted the disruptive effects of H_2_O_2_ and showed significant improvements in barrier integrity compared to the positive control. However, it is important to acknowledge that these in vitro findings were performed on adult human intestinal cells, which may not fully reflect the intestinal epithelium of neonatal rats. So far, there seems to be not much research discussing the effects of specific EVOO metabolites on the intestinal barrier, but there are many studies that state how EVOO improves gut health and mucosal immunity [56,57,58,59], highlighting the potential of this oil as a valuable component in maintaining intestinal barrier function. A study showed the ability of homovanilic acid esters to inhibit fatty acids in Caco-2 cells, suggesting a potential role in modulating intestinal function [60]. Although hippuric acid did not show significant effects in our study, a previous study on colitis suggests that it may have a positive impact on maintaining barrier integrity, improving its function and reducing inflammation [61].

## 4. Materials and Methods

### 4.1. Animals

Lewis rats (HanHsd), comprising 20 females and 5 males aged 8 weeks, were sourced from Envigo (Sant Feliu de Codines, Spain). The rats were housed in individual cages with free access to water and a standard diet formulated according to the AIN-93G guidelines [62]. For mating, each male was paired randomly with two females over a 48-h period, after which females were separated and individually housed. The day of delivery was established as day 1 of life. To standardize nutrition, each litter was adjusted to 6 pups per lactating dam, who continued to have unrestricted access to standard chow and water. Environmental conditions were regulated with a 12-h light–dark cycle, constant temperature, and humidity at the Animal Experimentation Unit, Diagonal Campus, Faculty of Pharmacy and Food Sciences, University of Barcelona. Ultimately, pups from 14 successful pregnancies were used at 21 days of age for experimentation.

All animal handling and procedures followed the guidelines outlined in the Guide for the Care and Use of Laboratory Animals. Ethical approval was granted by the University of Barcelona’s Animal Experimentation Committee and the Catalan Government (CEEA-UB Ref. 240/19 and DAAM10933, respectively), in full alignment with European Directive 2010/63/EU on animal welfare in scientific research.

### 4.2. Experimental Design

The dietary intervention was administered to the dams over a total period of 6 weeks—3 weeks of gestation followed by 3 weeks of lactation. Rats were randomly assigned to one of three treatment groups, receiving either extra virgin olive oil (EVOO), refined olive oil (ROO), or water. Treatments were administered daily by oral gavage at a dose of 10 mL/kg body weight throughout the intervention period. The EVOO, derived from the Picual variety, had a documented phenolic profile [28], with total polyphenol content of 862 mg/kg, predominantly consisting of oleuropein and oleacein. ROO served as the control oil group to distinguish the physiological effects of polyphenol content from those of dietary fat alone.

Offspring were monitored by daily weight measurement from birth until weaning. On postnatal day 21, pup length (nose-to-anus) was measured to calculate the body mass index (BMI) using the following formula: $body\;weight/{length}^{2}$ ($g/{cm}^{2}$). The Lee index was calculated as $\sqrt[{3}]{weight}/length\times 1000$ ($\sqrt[{3}]{g}/cm$).

### 4.3. Sample Collection and Processing

On the final day of suckling (d21), pups were anesthetized using a combination of ketamine (90 mg/kg; Merial Laboratorios, SA, Barcelona, Spain) and xylazine (10 mg/kg; Bayer AG, Leverkusen, Germany). Blood samples were obtained via cardiac puncture, collected in hematology-specific tubes (Spincell, Monlab Laboratories, Barcelona, Spain), and centrifuged to isolate plasma for immunoglobulin (Ig) quantification, which was subsequently stored at −20 °C.

A portion of the small intestine (mid-section) was dissected for further analysis. One section was preserved in RNA later at −20 °C for gene expression studies by PCR, another was used for Ig measurement, and a third was processed for histological evaluation. Additionally, mesenteric lymph nodes (MLNs) were excised, and the following organs were weighed: liver, spleen, thymus, small intestine, cecum, stomach, kidneys, and heart. The total length of the small intestine was also recorded.

### 4.4. Immunoglobulins Quantification

For immunoglobulin (Ig) analysis, between 12 and 15 pups per treatment group (approximately 3–4 offspring per dam) were selected. Plasma and homogenized mesenteric lymph node (MLN) samples collected on postnatal day 21 were analyzed for concentrations of IgA, IgM, IgG1, IgG2a, IgG2b, and IgG2c using the ProcartaPlex Rat Antibody Isotyping Panel (eBioscience, Frankfurt, Germany). The assay was conducted according to the manufacturer’s instructions and protocols previously described in the literature [63]. Sample acquisition was performed using a Luminex MAGPIX^®^ system (Luminex^®^, Austin, TX, USA) at the Flow Cytometry Unit of the Scientific and Technological Centers of the University of Barcelona (CCiT-UB).

The detection sensitivities of the assay were as follows: 0.48 pg/mL for IgA, 0.02 ng/mL for IgM, 0.78 ng/mL for IgG1, 0.02 ng/mL for IgG2a, 0.11 ng/mL for IgG2b, and 0.19 pg/mL for IgG2c. To assess immune polarization, Th1-associated isotypes (IgG2b + IgG2c) and Th2-associated isotypes (IgG1 + IgG2a) were aggregated as composite measures, following the approach detailed in previous studies [41].

Quantification of total cecal bacterial load and the proportion of immunoglobulin-coated bacteria (Ig-CB) was conducted using a method previously established in the literature [64]. Flow cytometry was carried out using a Cytek Aurora cytometer (Cytek Biosciences, Fremont, CA, USA) at the FCU of the CCiT-UB, with data acquisition capped at 25,000 events per sample. Data processing and analysis were performed using FlowJo version 10 (Tree Star Inc., Ashland, OR, USA).

### 4.5. Galectins, Adiponectin, and Leptin Quantification

The same subset of pups used for immunoglobulin analysis was also selected for the quantification of galectin (GAL), adiponectin, and leptin. GAL-3 and GAL-9 levels were measured using specific ELISA kits (Elabscience Biotechnology Inc., Houston, TX, USA). Adiponectin concentrations were determined with the ADP/Acrp30 ELISA kit (Elabscience Biotechnology Inc.), while leptin levels were assessed using the Mouse/Rat Leptin Immunoassay kit (R&D Systems, Inc., Minneapolis, MN, USA). All procedures followed the manufacturers’ protocols and were consistent with previously described methods [28].

### 4.6. Histology

A segment of the medial region of the small intestine was preserved in 4% paraformaldehyde for 24 h, followed by dehydration and paraffin embedding. Tissue blocks were sectioned at a thickness of 5 µm using an RM2135 microtome (Leica, Wetzlar, Germany) at the Microscopy Unit of the CCiT-UB. The resulting sections were stained using hematoxylin–eosin (H&E) and periodic acid–Schiff (PAS) techniques, mounted with ProLong™ Gold Antifade Mountant (Life Technologies, Carlsbad, CA, USA), and covered with glass slips.

Microscopic examination of the intestinal samples was carried out under bright-field illumination at 100× magnification using an Olympus BX41 microscope (Olympus Corporation, Shinjuku, Tokyo, Japan). Morphological assessments were performed using ImageJ (Version 1.54p; National Institute of Mental Health, Bethesda, MD, USA). The analysis included quantitative measurements of villus height, width, and surface area; crypt depth; the villus-to-crypt ratio; and the number of goblet cells per villus.

### 4.7. Gene Expression Study

For the gene expression study, nine pups per group (three per dam) were selected. Intestinal samples preserved in RNA later were homogenized for 30 s using lysing matrix tubes (MP Biomedicals, Illkirch, France) and the FastPrep-24 homogenization system (MP Biomedicals). Total RNA was isolated following the standard protocol of the RNeasy^®^ Mini Kit (Qiagen, Madrid, Spain). RNA concentration and purity were assessed with a NanoPhotometer (BioNova Scientific, CA, USA).

Complementary DNA (cDNA) was synthesized using the PTC-100 Programmable Thermal Controller and TaqMan^®^ Reverse Transcription Reagents (Applied Biosystems, Weiterstadt, Germany). Quantitative real-time PCR was then performed on an ABI Prism 7900 HT system (Applied Biosystems) using gene-specific TaqMan^®^ primers, as listed in Table S1. The glucuronidase beta (Gusb; Rn00566655_m1) gene was used as the endogenous reference for normalization.

Relative gene expression levels were determined using the 2^–ΔΔCt^ method. Data are expressed as percentages, with each treatment group normalized to the average expression value of the reference (REF) group, which was set at 100%, in line with previously established protocols [65,66].

### 4.8. In Vitro Experimentation

#### 4.8.1. Cell Culture

Per the usual protocol, Caco-2 cells (American Type Culture Collection, ATCC, Manassas, Virginia) were grown in plastic flasks in Dulbecco’s modified Eagle’s Medium (DMEM) supplemented with 10% fetal bovine serum (FBS) and 1% penicillin–streptomycin. Cultures were maintained in a 5% CO_2_ atmosphere, with the medium being refreshed every two days. When the Caco-2 cells reached 80–95% confluence, they were subcultured and reinoculated into a new 75 cm^2^ flask at a 1:3 dilution [67]. Cells (passage 21–33) were subcultured at a density of 10^4^ cells/cm^2^ on 12-well clusters (Costar, Cambridge, MA, USA) for the lactate dehydrogenase (LDH) experiment and at a density of 1.5 × 10^5^ cells/cm^2^ on polycarbonate filters (Transwells, with 1 µm pores and a surface area of 1.12 cm^2^) (Costar, Cambridge, MA, USA) for permeability experiments.

Cells were preincubated for 24 h at different concentrations for LDH (50–1000 nmol/mL) and for permeability experiments at 1000 nmol/mL with the three EVOO metabolites of interest based on previous studies [28]: HPP, HV, and HFA.

#### 4.8.2. Lactate Dehydrogenase Activity

The potential cytotoxic effect of EVOO compounds was evaluated by determining the relative activity of the lactate dehydrogenase (LDH) enzyme in the incubation medium using the LDH Cytotoxicity Assay Kit (BioChain Institute Inc., Newark, CA, USA) at a wavelength of 490 nm (Sunrise, Tecan, Grödig, Austria).

#### 4.8.3. Measurement of Trans-Epithelial Electrical Resistance (TEER)

After 24 h of incubation with the EVOO metabolites, cells were incubated with H_2_O_2_ (3 mM, 3 h), and TEER was measured as previously described [68] at the initial time before adding H_2_O_2_ and every hour after H_2_O_2_ incubation over 3 h. Changes in TEER during the experimental conditions were calculated as a percentage of the corresponding baseline values. Duplicate cell monolayers were used for each group in every experiment, and the experiment was repeated three times.

### 4.9. Statistical Analysis

All statistical procedures were conducted using the IBM SPSS Statistics software (v22.0, Chicago, IL, USA). To determine whether the data followed a normal distribution, the Shapiro–Wilk test was applied, while Levene’s test was used to assess the equality of variances across groups. When both assumptions—normality and homoscedasticity—were satisfied, one-way analysis of variance (ANOVA) was used. In such cases, the Bonferroni post hoc test was employed for pairwise comparisons if variances were equal, or the Dunnett test when they were not. For datasets that did not conform to normal distribution, the non-parametric Kruskal–Wallis test was utilized, followed by Dunn’s test for post hoc comparisons. A threshold of *p* < 0.05 was considered indicative of statistical significance in all analyses.

## 5. Conclusions

Overall, our current research reveals that maternal EVOO and ROO supplementation influences the offspring’s immune and intestinal health. Even though both oils have an impact on offspring, EVOO seems to provide specific benefits, particularly in immune modulation, probably due to its phenolic compounds. The increase in both IgG2c and *FcRn* expression shows the potential role of EVOO in promoting immune system development. These findings further support the accumulating evidence on how maternal dietary interventions benefit offspring health.

## Figures and Tables

**Table 1:** Morphometric variables and relative organ weights or lengths at the end of the suckling period.

	REF	ROO	EVOO
Body weight (g)	32.69 ± 1.50	28.77 ± 0.61 *	27.10 ± 0.71 *
Naso-anal length (cm)	10.51 ± 0.17	10.29 ± 0.07	9.93 ± 0.12 *
BMI (g/cm^2^)	0.29 ± 0.01	0.27 ± 0.01 *	0.27 ± 0.01 *
Lee index	303.10 ± 2.01	297.46 ± 1.46	302.40 ± 1.60
Stomach (%)	0.82 ± 0.02	0.88 ± 0.02 *	0.88 ± 0.01 *
Liver (%)	3.43 ± 0.09	3.66 ± 0.1	3.30 ± 0.05 #
Spleen (%)	0.29 ± 0.01	0.28 ± 0.01	0.28 ± 0.01
Thymus (%)	0.45 ± 0.02	0.41 ± 0.01	0.46 ± 0.02
Right kidney (%)	0.71 ± 0.01	0.67 ± 0.01	0.68 ± 0.01
Heart (%)	0.72 ± 0.01	0.97 ± 0.26	0.70 ± 0.01
Caecum (%)	1.34 ± 0.07	1.47 ± 0.1	1.16 ± 0.08
Small intestine weight (%)	3.52 ± 0.08	3.86 ± 0.11 *	3.55 ± 0.05 #
Small intestine length (%)	117.38 ± 5.53	129.63 ± 3.14 *	127.90 ± 3.40 *

Morphometric variables are expressed as mean ± standard error of the mean (SEM) (n = 18–36 pups/group). BMI: body mass index. Organ weights expressed in g of tissue/100 g of body weight. Organ length expressed in cm of tissue/100 g of body weight. Statistical differences: * *p* < 0.05 vs. REF # *p* < 0.05 vs. ROO.

**Table 2:** Hematological variables at d21 of the suckling period.

	REF	ROO	EVOO
Leukocytes (×10^9^/L)	2.38 ± 0.65	1.78 ± 0.37	1.33 ± 0.18
Lymphocytes (×10^9^/L)	1.78 ± 0.52	1.13 ± 0.23	0.91 ± 0.12
Monocytes (×10^9^/L)	0.08 ± 0.03	0.07 ± 0.05	0.02 ± 0.01
Granulocytes (×10^9^/L)	0.52 ± 0.11	0.58 ± 0.10	0.40 ± 0.05
Lymphocytes (%)	75.35 ± 1.29	66.65 ± 2.15	71.63 ± 1.92
Monocytes (%)	5.88 ± 0.52	6.45 ± 1.08	6.29 ± 0.44
Granulocytes (%)	18.77 ± 1.24	26.91 ± 1.68	22.08 ± 1.65
Platelets (×10^9^/L)	217.67 ± 84.09	217.33 ± 79.17	183.56 ± 86.86
Erythrocytes (×10^9^/L)	3.73 ± 0.42	4.75 ± 0.19 *	3.94 ± 0.26 #
HGB (g/L)	70.17 ± 8.41	84.01 ± 2.38	65.22 ± 5.12 #
HCT (%)	20.98 ± 2.33	26.02 ± 0.91 *	21.63 ± 1.48 #
VCM (fL)	56.53 ± 0.48	55.03 ± 0.72	55.10 ± 0.56
HCM (pg)	18.68 ± 0.65	17.70 ± 0.35	17.38 ± 86.86

Results are expressed as ± S.E.M (n = 18–36). * *p* < 0.05 vs. REF group # *p* < 0.05 vs. ROO group.

## Data Availability

The original contributions presented in this study are included in the article/Supplementary Material. Further inquiries can be directed to the corresponding author.

## Supplementary Materials

The following supporting information can be downloaded at https://www.mdpi.com/article/10.3390/ijms26167946/s1.

## Abbreviations

The following abbreviations are used in this manuscript:

BW	Body weight
EVOO	Extra virgin olive oil
HCM	Mean corpuscular hemoglobin
HCT	Hematocrit
HFA	Hydroxyphenylacetic acid
HGB	Hemoglobulin
HPP	Hippuric acid
HV	Homovanillic acid
Ig	Immunoglobulin
IS	Immune system
LDH	Lactate dehydrogenase
REF	Reference
ROO	Refined olive oil
TLR	Toll-like receptors
TEER	Trans-Epithelial Electrical Resistance
VCM	Mean corpuscular volume
