# Supplementary figures and images for: Maternal Extra Virgin Olive Oil Supplementation Enhances Offspring Immune Function: A Preclinical Study
Source: Int J Mol Sci. 2025 Aug 18;26(16):7946. doi: 10.3390/ijms26167946 (PMC12386658; doi:10.3390/ijms26167946)

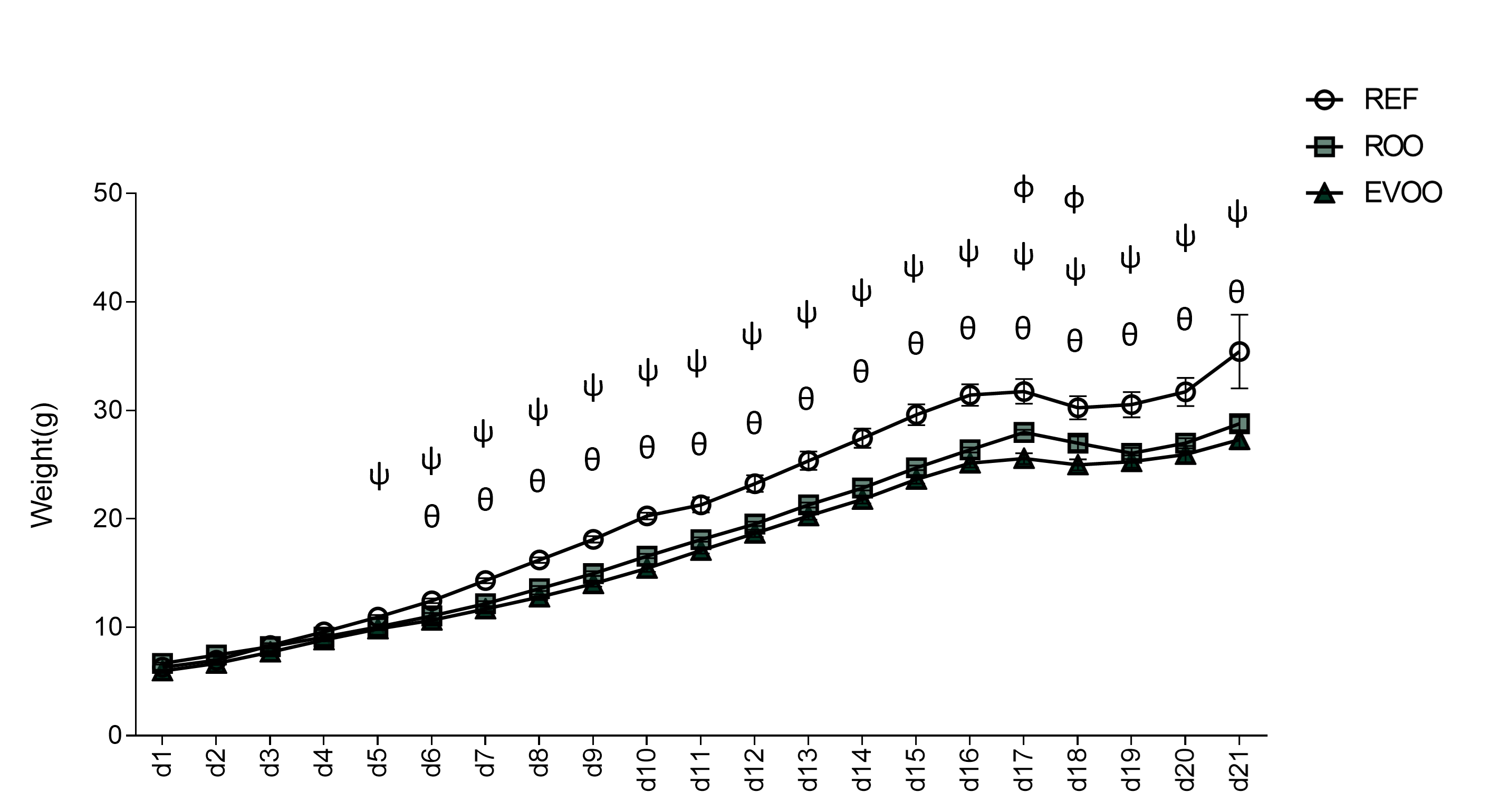

Supplement: Supplementary file 1 [file ijms-26-07946-s001.zip › Figure supl 1.png]

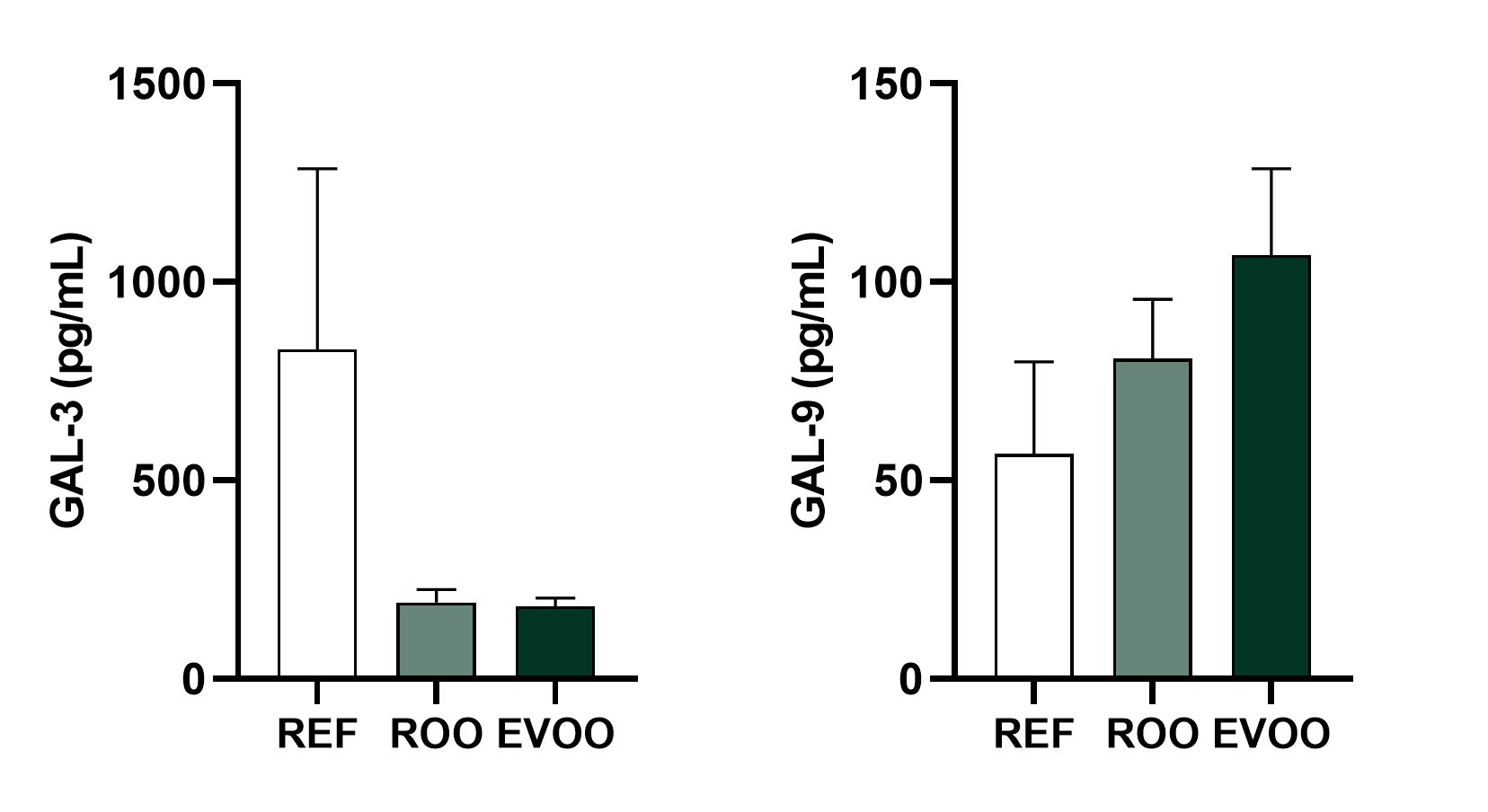

Supplement: Supplementary file 1 [file ijms-26-07946-s001.zip › Figure supl 2.png]

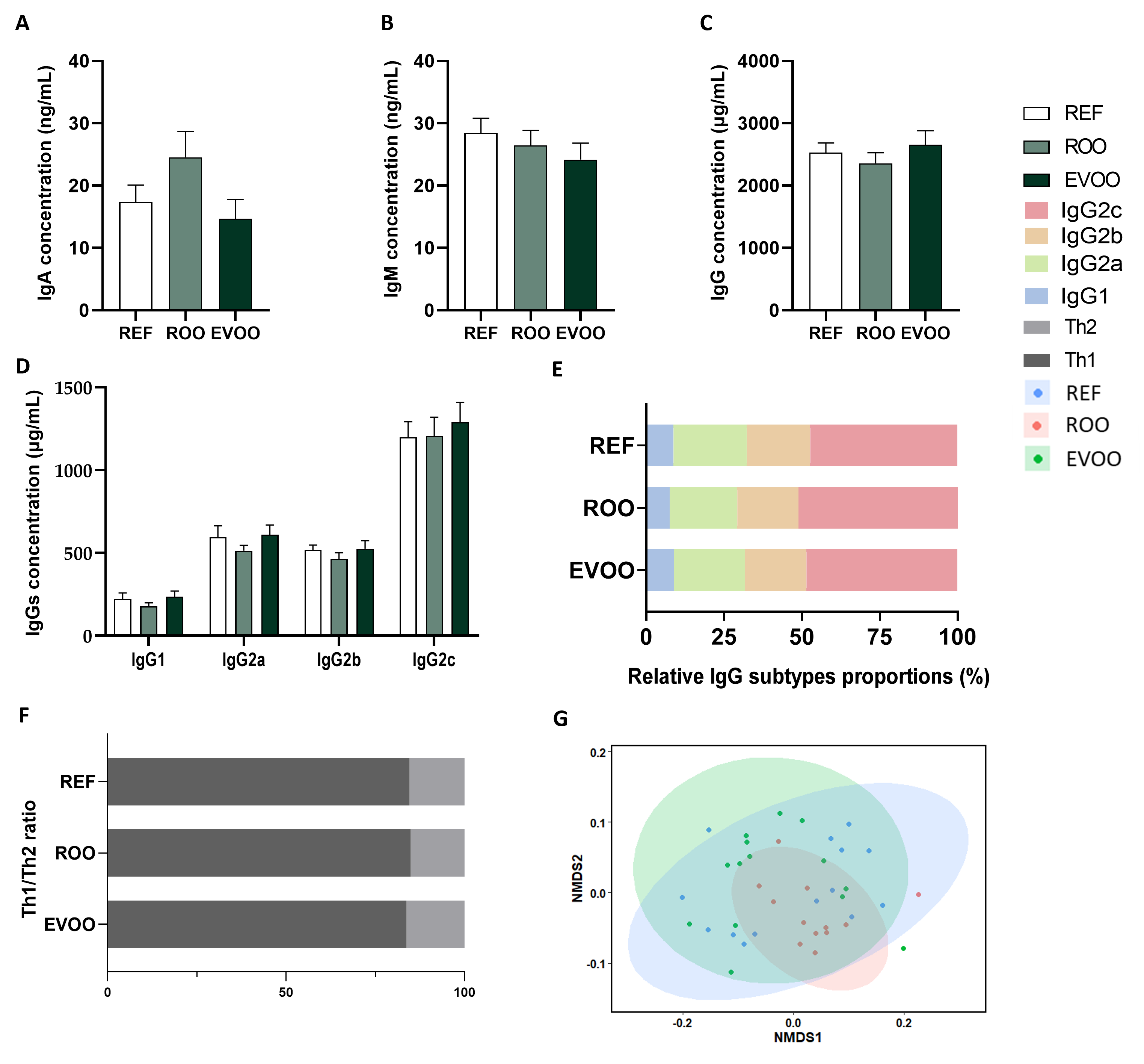

Supplement: Supplementary file 1 [file ijms-26-07946-s001.zip › Figure supl 3.png]
